# Ellen P. Reese: Always Give the Learner the Opportunity to be Right

**DOI:** 10.1007/s40617-025-01066-9

**Published:** 2025-05-21

**Authors:** William L. Heward, Jonathan W. Kimball

**Affiliations:** 1 College of Education and Human Ecology, The Ohio State University, Columbus, OH USA; 2 Behavior Development Solutions, Bonita Springs, FL USA

**Keywords:** Animal rights/welfare, Experimental ethics, Omission training, Operant conditioning, Rule-governed behavior, Teaching

Ellen Reese (1926–1997) spent her entire adult life at Mount Holyoke College in South Hadley, Massachusetts. She earned a BA in 1948, her MA in 1954, and was named the Norma Cutts Dafoe Professor of Psychology in 1994. Mount Holyoke College dedicated the Ellen and Thomas Reese Psychology and Education Building in 1996.

Ellie, as she preferred to be called, studied ethology and published research in comparative psychology. She designed and directed animal research laboratories, wrote experimental manuals, and produced instructional films. Her 1965 film, *Behavior Theory in Practice*, was translated into over 40 languages by the United States Information Agency, thereby introducing thousands to basic and applied behavioral research (Morris, 1998). *Born to Succeed* (1970), a film featuring one of her honors students using fading to teach number concepts to a student with intellectual disabilities, is a classic.

In addition to publishing scholarly articles and chapters across five decades, Professor Reese authored the widely used textbooks *Experiments in Operant Behavior* (Reese, 1964) and *The Analysis of Human Operant Behavior* (Reese, 1966). Of her monograph, *Human Operant Behavior Analysis and Application* (Reese, 1978), Beth Sulzer-Azaroff (2017) wrote, “Ellie had elegantly distilled the methods and numerous findings of behavior analytic investigations in as compact, lucid, and appealing a way as anyone had to date” (p. 156).

First and foremost, Ellie was a teacher who influenced two generations of behavioral scholars. “As a testament to her impact as a mentor, an astounding 35 of her undergraduate students went on to earn doctorates... Given that students are, and always have been, the future of behavior analysis, our discipline is today much of Ellie’s making” (Morris, 1998, p. 141). In an appreciation published shortly after Reese’s death, Judy Favell (1997) wrote:

Her presence was always felt. With her spare, straight frame and handsome face (usually bracketed by brightly colored animal earrings), she was never intentionally obtrusive but nevertheless was unfailingly noticed. At conventions and other events, both the budding and the blossomed alike would point her out, greeting her with affection and respect. She deserved such regard. (p. 723).

Her films, texts, manuals, and workshops represent the best in teaching principles of behavior, from their basic dimensions to their operation in everyday lives. Her demonstrations of conditioning across species, settings, and circumstances remain among the most inspiring and essential lessons on the power of these principles. She devoted her career to teaching these foundations from which all behavior-analytic work emanated, and shared concerns when these roots were disregarded or minimized. (p. 724).

**Photo 1:**
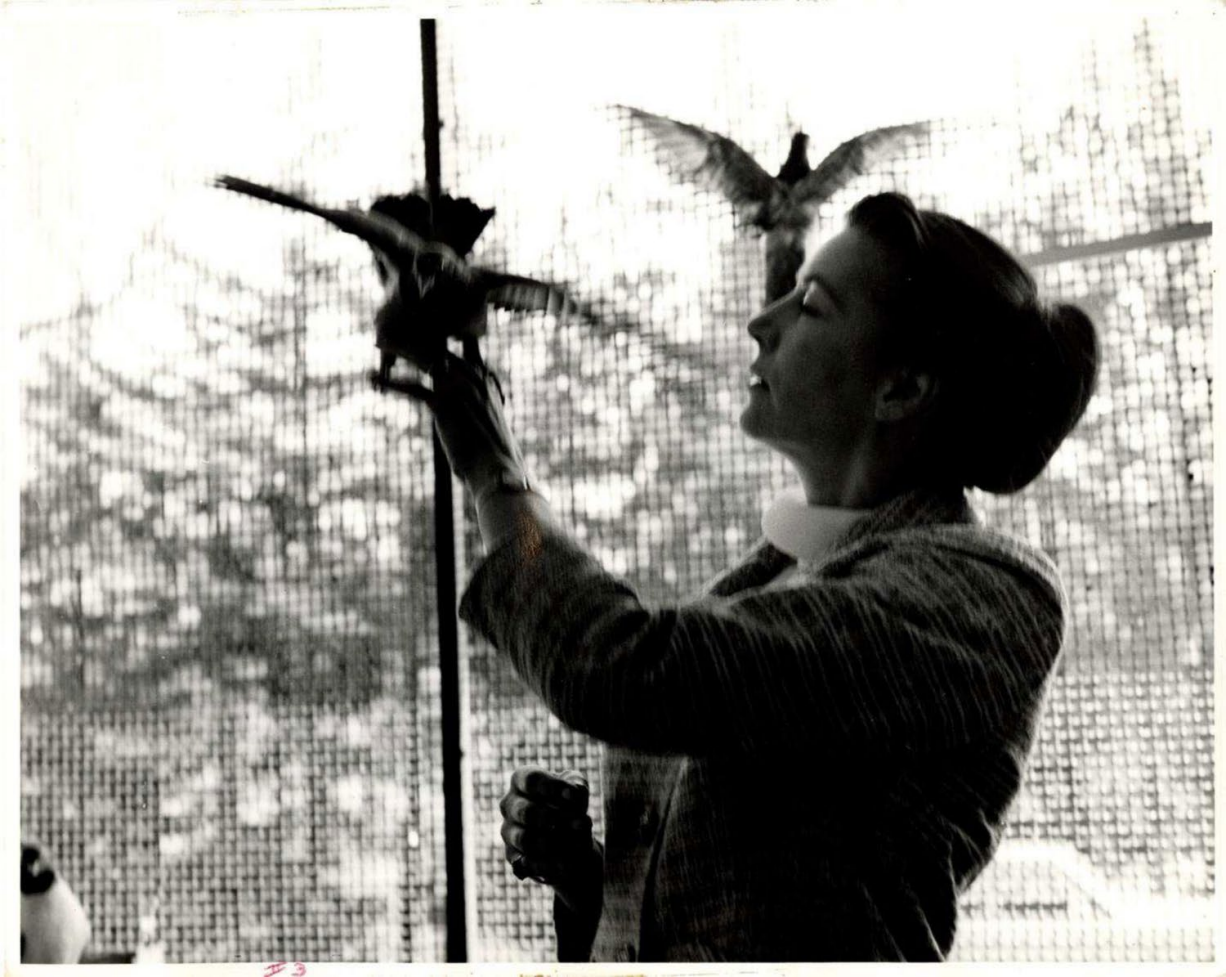
Ellen P. Reese (1926–1997). **“**I love animals.” (Photo courtesy Mount Holyoke College Archives and Special Collections)

Ellen Reese held prominent leadership positions in behavior analysis and psychology, serving as president of the Association for Behavior Analysis International and Division 25 (Behavior Analysis) of the American Psychological Association (APA). She received APA’s Distinguished Contribution to Education in Psychology Award in 1986 and is included in APA’s “100 Most Important Women in Psychology.” Photo 1

On November 7, 1991, Ellen Reese served as Distinguished Guest Faculty for Ohio State University’s Teleconference Seminar on Applied Behavior Analysis (Heward, 1989; Peterson, 2024). In preparation, the 20 students and faculty members who participated^1^ read “Behavioral Procedures for Assessing Visual Capacities in Nonverbal Subjects” (Reese et al., 1977) and “B. F. Skinner’s Contributions to Animal Welfare” (Reese, 1991), a manuscript that was under review by the *American Psychologist* for possible inclusion in a special volume dedicated to the work of B. F. Skinner, who had died in August 1990.^2^ This article was developed from an audio cassette recording of the 2-h telephone discussion with Professor Reese.

Professor Reese’s attention to student learning was evident throughout the teleconference. On several occasions, she read from a journal article or book by other authors to underscore a key point or provide an additional example of a concept she was explaining. Professor Reese was conversational, straightforward, and gracious. It was a master class in scholarship and humility.^3^ We hope this article will contribute to readers’ understanding and appreciation of the historical foundation of behavior analysis to which Ellen Reese was such a vital contributor and on which today’s behavior analysts continue to build a progressive science.

## I Thought Everyone was a Behaviorist

REESE: Hi, Bill. How are you doing?

HEWARD: Great Ellie. You're coming through just super. Can you hear me all right?

REESE: Yes. How many students are there?

HEWARD: Fifteen Ph.D. students, ranging from their first to third year of full-time study. They bring varied backgrounds to the program and some impressive professional experiences. The students have prepared questions based on the readings you assigned. Each student will briefly introduce themselves before asking their question. But first, I’ll begin with a question we ask each of our guest faculty. Would you describe the events in your life, academically or personally or otherwise, that led you to become a behavior analyst?

REESE: It was lot of luck. I started out as a zoology major. I came to Mount Holyoke because it probably had the best undergraduate zoology department in the country. I love animals, and I like to play with animals. And I thought, gee, as a zoology major, that’ll be great. I took a psych class freshman year and ended up pitching all my notes. I didn’t think they were worth much!

Then I took developmental psych, and that was a little better. In my junior year, I took experimental psych. Mount Holyoke had the very first behavioral lab program, along with Columbia—we even had Keller and Schoenfeld’s lab manual (1949) as a pre-published manuscript.^4^ Anyway, we had the first rat lab along with Columbia at the same time. And *that* was fascinating! Controlling somebody’s behavior! It was awesome.

So, then I decided I’d take some more psychology. And finally, senior year, I switched my major to psychology because there were more opportunities to work with vertebrates, at that time, than in zoology, which is now biology. And then I stayed on to work on an Air Force contract in psychophysics. That was my first training. My first publications were in that area.^5^ And I was teaching the animal lab. And then I married my boss.

HEWARD: Was that the lucky part, Ellie?

REESE: Yeah! And well, almost as a honeymoon, Tom had a Fulbright in Turkey where we set up the first psychology department experimental labs over there. And that’s how I got on the pigeons because we didn’t see how we’d ever get rats in Turkey.

They had pigeons everywhere in Turkey, so the kids caught their own pigeons in the park and made cardboard apparatus. The pigeons learned so much faster than the rats had, so when we got back to Holyoke, we switched over to birds.

Tom was almost 20 years older than I, and he knew all the early people. So, I just grew up knowing Fred Skinner and Fred Keller and all those people. And I just thought everyone was a behaviorist. It took me a while to realize that wasn’t the case.

**Photo 2:**
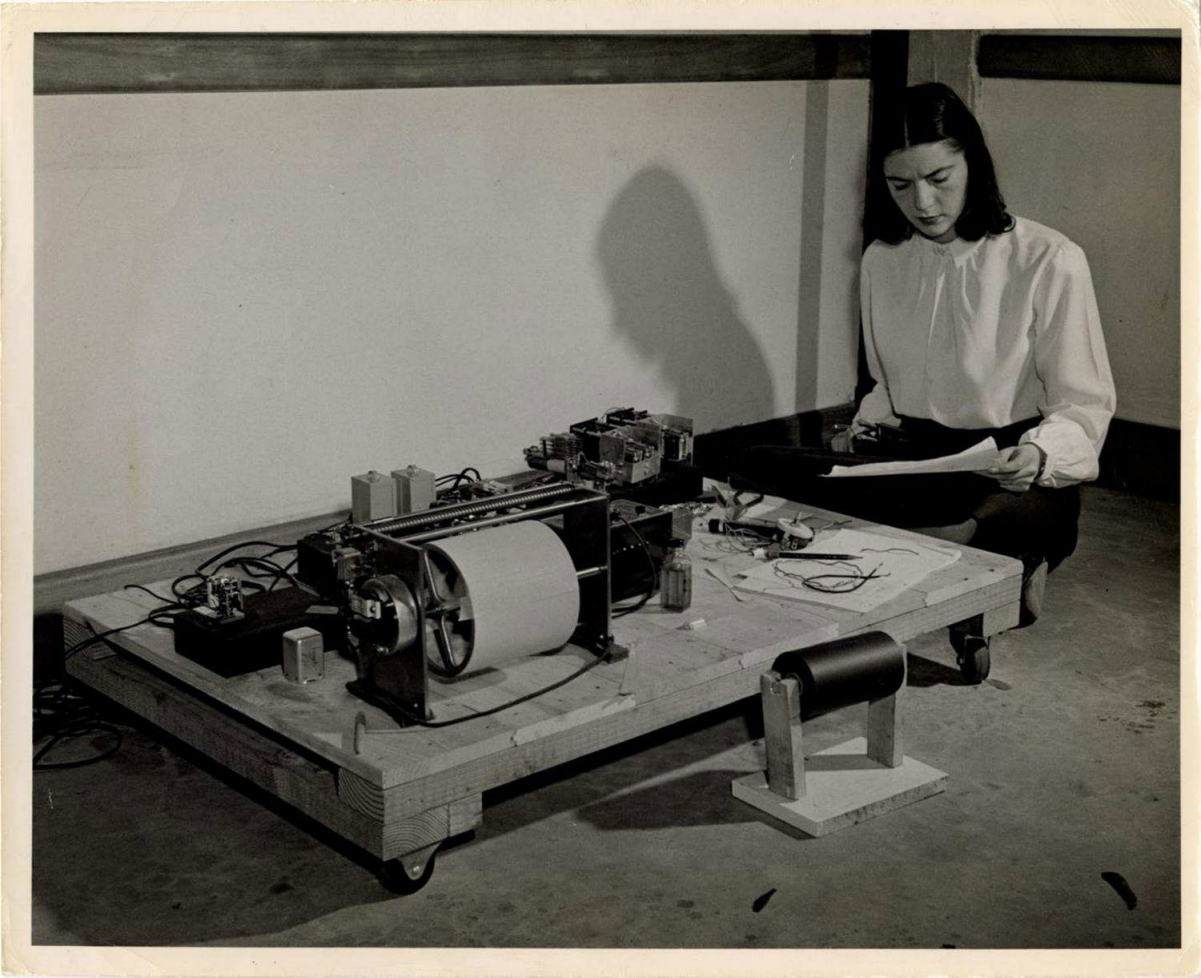
Ellen P. Reese constructing research apparatus in experimental laboratory at Mount Holyoke College. (circa 1950). (Photo courtesy Mount Holyoke College Archives and Special Collections)

HEWARD: That’s quite a story. Let’s turn it over to the students now.^6^ Photo 2.

## Correcting Misconceptions about Skinner

OSU: My question concerns the article “B. F. Skinner’s Contributions to Animal Welfare.” While efforts to improve the welfare of captive animals is commendable, I was just wondering whether the paper will help or hinder the perceptions of our field. One of the criticisms of behavioral approaches to teaching is that they’re manipulative and unfeeling and so forth, and that while behavioral techniques tend to work, the cost in terms of freedom and dignity is perceived by critics as too great. For example, teaching an animal to voluntarily submit to a medical procedure that it would otherwise avoid seems to be something that someone might construe as a contradiction between what a behavior analyst says they are doing as far as improving the welfare of captive animals and what they really are doing.

Another criticism is that behavioral techniques are fine for rats and pigeons and individuals with serious disabilities. But a lot of people would like the behavior analyst to leave the regular folks alone. Do you think this article will perpetuate or discourage those kinds of criticisms?

REESE: The assumption here is that laboratories are going to do these biomedical procedures anyway. I’m trying to get them to do that work more humanely.^7^ Now, that’s a different question from whether we should use animals in research at all.

OSU: The essence of my question isn’t whether or not we should. I guess a paper in the *American Psychologist* would be read by a very wide audience. I was just wondering, in general, about your thoughts as to whether or not the paper will improve perceptions of our field.

REESE: Well, I’m hoping it’s going to help. I picked this particular area hoping to correct misconceptions that Skinner advocated punishment, that he put his daughter in a box and presumably shocked her, that she wound up in a mental institution, and so on. I wanted to both correct those misconceptions and then show how we can do so much more than we are doing to benefit animals who are confined to labs.

Now, on the “behavior analysis is fine for rats,” the best way I know to handle that one—aside from reading Skinner’s answers to his critics, which are at the end of *About Behaviorism* (Skinner, 1974)—is to have people do self-management projects. Not only are we all controlling each other’s behavior all the time anyway, but we’re usually misusing these procedures. It’s awfully important that we learn how to control our own behavior, and self-management procedures, particularly stimulus control, will enable us to do that.

I know no answer to freedom except, again, Skinner’s answer that by developing competence in people, we give them options. We give them freedom to choose the way they will gain their reinforcers, whether it’s being able to read and enjoy a book or doing the work they want to do or whatever. Let me read you a quote from *The Technology of Teaching*.

It could well be that an effective technology of teaching will be unwisely used. It could destroy initiative and creativity; it could make men all alike (and not necessarily in being equally excellent); it could suppress the beneficial effects of accidents on the development of the individual and upon the evolution of a culture. On the other hand, it could maximize the genetic endowment of each student; it could make him as skillful, competent, and informed as possible; it could build the greatest diversity of interests; it could lead him to make the greatest possible contribution to the survival and development of his culture. (Skinner, 1968, p. 91).

## Always Provide the Opportunity to be Right

OSU: In the article on the behavioral procedures for assessing visual capacities, you referred to emotional behavior in the S-delta condition. I was wondering about the nature of emotional behavior observed in the other conditions.

REESE: There was none in the other conditions. But it was terrible in the extinction condition. Jane Howard—she’s marvelous; Janes’s out at Cal State Stanislaus now—she called me and said she couldn’t continue this experiment. These were great big guys. They were the people that Sidman and Stoddard worked with. They were cursing, banging the wall, and breaking the place apart. Jane couldn’t bear to see them so frustrated. I persuaded her to keep with the reinforcement-extinction paradigm. You know, that was really easy for me to say, sitting back here in South Hadley while she was up there. But she stuck it out. Even with people whose disabilities were less severe, we still saw a fair amount of emotional behavior, which is why I started studying the physiological problems of errors later on.^8^

We’ve been using Chinese Mandarin characters as our visual stimuli with college students as subjects in studies looking at the physiological toll of errors. I picked those characters because they were hard, and everything else is too easy for college students. We use a match-to-sample task with six alternatives. If we project the pattern for 5 s, we get enough errors to study. And they’re very detrimental, the errors are. Unless we change the instructions and point out that errors are an opportunity to learn. By using that instruction or something like fading, we can greatly improve performance on transfer tasks and follow-up.

We don’t see emotional behavior when there’s an opportunity to be right and during reinforcement. I’ve never seen serious emotional behavior with fading. And the funny thing is we were trying to use fading with the reinforcement-extinction group. But we couldn’t even get going. Emotional behavior occurred before we even presented the stimulus. But fading requires a fair amount of work to establish the stimulus materials. For kids who can’t learn any other way, fading is wonderful. But I don’t think you’re going to get terribly many teachers to use fading unless we can develop programs that schools would buy.

The thing is, most kids learn despite ineffective teaching. A very good article on that was written by Barbara Etzel and Judy LeBlanc (1975). Their point is that you really adapt the procedure to the needs of your particular kids. If the kids can’t learn with simpler programs or more ordinary programs, get increasingly more supportive with programs like fading and stimulus shaping. Their recommendations apply to any class. Etzel and LeBlanc’s paper ought to be required reading if you’re getting into teaching.

## Shape Up Some Constructive Behavior

OSU: There’s a certain amount of controversy on managing aggressive behavior that presumably functions as escape.

REESE: The Ed Carr and Mark Durand stuff?^9^

OSU: Yes. The complaint is that when you use functional communication training as opposed to escape extinction, you tend to have very low productivity, to the point at which there’s a trade-off of getting less problem behavior with FCT but the learner making few, if any, task-related responses.

REESE: Well, yeah. I mean, nobody’s going to buy that. If you can locate the reinforcer, you want to extinguish the problem behavior. But at the same time, you have to be shaping up some constructive behavior. And that seems to eliminate the detrimental effects of extinction because there’s plenty of reinforcement coming along. One thing I like about Carr and Durand is that we’re finally trying to look at the context in which behavior is occurring and identifying the reinforcers. And then you know whether it’s escape, avoidance, or maybe there’s even a medical problem.

## Aversives Complicate Things

OSU: I’m interested in the role of aversives in rule-governed behavior. Malott suggests that a rule statement is an establishing operation that establishes non-compliance with the rule as an aversive condition.^10^ For example, he suggested if you state a rule such as, “If I don’t start reading this chapter, I’m not going to be ready for the quiz,” your procrastination produces an aversive condition, a respondent people might call guilt. As time for the quiz grows close, you rush to get the work done. Stating the rule is analogous to turning on the shock in the escape experiment and studying for the quiz analogous to an escape response. A poor grade on the quiz or even good performance on the quiz is too delayed to be a direct acting consequence for the behavior of studying.

My question: If we look at human animals, rule-governed behavior can create an aversive establishing operation. Perhaps what we need is more aversive EOs, because we have so many undesirable delayed consequences that can’t compete with strong reinforcing consequences operating in our environment at present. What are your thoughts on that?

REESE: I do think Dick Malott is the most thought-provoking person. I have a little trouble with this one. I think one reason we’ve evolved to write rules is to bridge the delays in consequences. In my experience with both animals and people, aversives always result in respondent conditioning in addition to their roles in suppression or avoidance of operant behavior. And that complicates a lot of things. That’s how it gets into guilt, I think. Anxiety is the more usual one. But I don’t think I’m answering your question. What was the question again?

OSU: We can generate aversive conditions to make ourselves avoid, and that’s a useful tool in both education and also just in our daily lives. I’m wondering, with what you know about aversives, are all aversives bad? Because it seems to me when we look at some of the large issues in the world, what we’ve got is control by immediate consequences, many of which, in the long term, are not good for the survival of our species.

REESE: I think we’re so busy generating aversive consequences for all life on the planet that I think it’s terribly important to specify what those aversive consequences are. Malott’s question of delayed control is one of the unfortunate things we’ve discovered in behavior analysis and the reason we have to develop these bridges. There are terrifying films on television of dolphin killing or oil spills or the oil wells burning in Kuwait. I think they help point out what some of these consequences are, but those images may not be effective because we’re not really *in* that situation.

And that’s why I like role-play of the kind that students do when they’re trying to get money for Oxfam.^11^ They’ll have meals, and they eat only rice, and the rest of the school’s money then goes to Oxfam. But they’ll have whatever proportion it is of the world, say 10% that has plenty to eat, get a very fancy dinner, and they were sitting there, and they had to eat it, and they were embarrassed as hell while all their colleagues were eating nothing but rice and some gruel. And that seems to bring the contingencies a little closer to home. And maybe we could develop more scenarios like that to try and bring some of these delayed consequences a little closer to home. Is that any kind of answer?

OSU: It’s helpful. My general sense is that aversives, in the sense of the rule-governed behavior example I gave, really need to be programmed across time. Otherwise, they’ll generate too much counter-control, and you’ll lose the battle in the first place. Or secondly, they’re very short-lived, which is probably a larger concern that I have. It would be interesting to see, for the Oxfam example, whether the treatment lasted and for how long.

REESE: Okay. But I do think we need to find out what makes a rule effective rather than some of the arcane work that’s coming out. At least to me, it’s arcane. It’s very difficult to read. Maybe somebody should play around with different kinds of rules and see how long-lasting some of them are. That would make rule-governance research a lot more interesting for me to read.

## The Marvelous Abilities of Animals

OSU: My question is about something in the media today. People are protesting the training and use of animals in entertainment, such as circuses and aquatic shows. Even the sexual habits of the animals have been dictated by man. For example, here in Columbus, we have a gorilla being shipped to another zoo which has several fertile females, because the female that he was starting to become friendly with here in Columbus is infertile. Why should these animals, such as the majestic elephant, be trained to stand on its hind legs, or a killer whale jump in the air on command? Is it the human’s ego insisting that our superior cognitive ability gives us dominion over these lower animals?

REESE: Let’s take zoos and aquaria rather than rodeos. There are programs that are reasonably good, like Karen Pryor’s at SeaWorld in Hawaii. There are two sides of it. There are some data, not anywhere near enough, but the main reason that zoo caretakers will give you for having animals confined in captivity, aside from preserving the species, is education of the public. And there are some data that children who go to zoos and see some of these programs then contribute more to animal welfare funds and conservation stuff. I think there’s some data on their reading, too: They’re more apt to read about animals than kids who haven’t had this experience.

The best teaching of behavior modification I have ever heard was either the San Diego aquarium or the LA aquarium, and they were putting sea lions and dolphins and orcas through their paces and describing what they were doing. Now—and it’s also true with Lipizzaner horses—they do not train any of those animals to do anything that they don’t do in the wild. And sometimes we don’t appreciate that because we don’t know all the magnificent things they do in the wild. We do train them to do it on our command rather than their own command. But it’s not like teaching a parrot to ride a bicycle, which it never would do in the wild; it doesn’t even do anything that approximates that.

It bothers me that orcas are in captivity, and dolphins, when they don’t look in good condition. If the dorsal fin is flopping over, that’s a sign of stress. And they don’t live as long in captivity. Those things bother me a whole lot. I don’t think they have enough room. The San Francisco Aquarium that’s right in Golden Gate Park has dolphins in a tank that’s much too small for them to really get up to speed and have a good time and exhibit their natural repertoire. But that’s not true all places. I think you have to look at the separate programs.

Our egocentric speciesism is certainly an issue, because we don’t know much about the abilities of other animals. I’m all for education in that respect. In Los Angeles, they took a red-shouldered hawk up in a helicopter and then let it fly down, and it went into a gorgeous stoop and landed on the handler’s wrist on the ground. And they were explaining the wonders and the marvelous abilities of all these animals.

OSU: Were you aware of the example that I mentioned concerning the gorilla being moved?

REESE: Well, I know they do that a lot. I think I would care more about how they handled him. Did they have to knock him out, chemically, which they probably did, to move him?

OSU: I know several of the animal rights groups were upset by the fact that it had taken several years for the gorilla to form an attachment to a female, and the fact that she was infertile led the zoo officials to believe that they needed to remove him from the setting where she was and put him in a setting where there were fertile females, and they never attempted to do this until after he started becoming friendly. And their objection was that…

REESE: Do you know how old he was? Because they take a long time to reach sexual maturity. You know, if there is a serious shortage of gorillas, which there certainly is in the wild, maybe send them over and let them impregnate several of the females in a colony—they’d have a nice time—and then bring them back. That’s what I would recommend.

## They Didn’t Have the Skills to Earn a Living

OSU: In your article, “B. F. Skinner’s Contribution to Animal Welfare,” you mentioned Skinner’s belief that aversive procedures should be replaced whenever possible with positive reinforcement. Could you share your position on how this approach might be applied to prison reform?

REESE: Cohen and Filipczak described how this could be done in the federal penal system.^12^They were at the National Training School in Washington, D.C., working with young prisoners who were there for all kinds of things, I mean, rape and murder. It was a beautiful educational program, a token economy, and they went through their high school program. They had to pay to enter a course. They earned points or tokens or something for all kinds of good behavior. The philosophy was that the reason these kids were criminals and would continue to be criminals once they did get out was that they didn’t have any skills that would enable them to earn a living by more acceptable means. And so it was really an educational program. It was in effect for at least three years. I spent a day there once, and it was one of the most exciting times I’ve ever had. And then it got taken over by some social workers who didn’t buy the behavioral part of the program. And, of course, that was key to the program.

Participants could buy anything they wanted out of the Sears Roebuck catalog, after the guns and knives pages were taken out. And they ended up buying clothes, like the clothes the teachers wore. Some of them, instead of putting up girlie pictures, began putting up classic art. They were also allowed to earn private enclosures for their bedrooms. It was just a marvelous program. I don’t remember anything aversive except response cost. And that’s not very aversive if you’ve got plenty of tokens. If somebody was completely without tokens, they went on what was called welfare, and then that was just like the normal prison. I think our prisons are so terrible now; I can’t imagine working with one. Do you know anything about them?

OSU: No more than what you’ve already said. And it seems the only thing we’re doing is that we’re building more. We’re not improving the system, it would appear.

REESE: And it gets doubly complicated when there’s an involvement with drugs. Obviously, those people ought to be in a rehabilitation program. Many of them want rehabilitation programs, but that isn’t where we put the money. We put the money in helicopters and airplanes between Florida and Columbia. And the rate of return on that is something like $1 of cocaine recovered for, I don’t know, $10 million spent on personnel and airplanes. The program, apparently, is a complete flop, and yet we’re putting more money into that than we are into rehabilitation programs. Now I’m depressed!

## Look for Good Behavior and Reinforce It

OSU: You mentioned that omission training has been used to eliminate behavior that’s well-established in an individual’s repertoire. Could you give us some examples of how omission training procedures can help reduce student’s problem behaviors?

REESE: A strict omission contingency would be, if there’s no disruptive behavior for 10 min or 5 min or a half an hour, then everybody gets points toward something or other. You’re reinforcing the absence or the omission of the problem behavior. If these are little kids, you don’t want them too stiff and rigid—they can horse around a little bit.

Anytime you’re using any reductive procedure, whether it’s extinction or punishment or omission training or any of the rest of them, you still want to make sure that people reinforce appropriate behavior. But by and large, we don’t notice appropriate behavior. We only notice inappropriate behavior because it annoys us. When everything’s going well, it wouldn’t occur to me to tell one of my college classes, "Oh, aren’t you wonderful? You’re all paying attention. You’re not running around the room, or burping, or whatever." So, sometimes, you have to train teachers and attendants to look for good behavior and to reinforce it. But omission training is a nice procedure. And it’s very flexible because as kids learn self-control, you can gradually increase the time they have to be good before they earn the reinforcer.

## Opportunity to Work for Reinforcers Makes Happier and More Active Animals

OSU: My question is from your “B. F. Skinner’s Contributions to Animal Welfare” article. In your review of “freeloading”,^13^ as opposed to working for food, you make a statement that “even in the presence of companions, the opportunity to work for food and other reinforcers appear to make for happier as well as more active animals.” Do you think this has implications for the way our social safety nets operate?

REESE: I think it’s a terrible mistake to do everything for somebody if they can do it for themselves. The person who taught me that was Jane Goodall. I had a pet grackle that I couldn’t release because he was injured, and I was preparing all his food for him and everything. And she pointed out that I was doing him a disservice because he’d be much happier foraging, getting peanuts out of the whole shell, and things like that. I think any time people can earn their reinforcers and control their environment that way, it’s just extremely beneficial. I think freeloading, whether it’s for food or for entertainment or anything else, just isn’t as satisfying.

And there is a little people research on this.^14^ They’re essentially letting people choose what they want to do and what they want to work for. Participants choose the task and the reinforcer. Now, that’s maybe a little tangential to what you’re saying, but it really decreases self-injury, aggression, all those unfortunate things. And it makes perfect sense to me.

## Research Ethics

OSU: My question is an offshoot of your article, “B. F. Skinner’s Contributions to Animal Welfare.” I was wondering what your impressions are of experiments that place human subjects under sometimes severe emotional stress. Specifically, a study that I think many people are familiar with, the Milgram Obedience Studies.^15^ Are you familiar with that?

REESE: I think Zimbardo is even worse.^16^

OSU: My advisor in my master’s program was a student of Milgram’s. The study seemed to yield some useful data, but it’s been universally condemned as being unethical and putting people through a lot of stress.

REESE: Well, it probably wouldn’t be accepted now, except that I think most review boards would assume people would stop delivering shocks rather than keep going.

OSU: Ethically, is there a place in psychology for conducting experiments that put people under severe emotional stress? And if there is, how do you draw a line, and how is your debriefing, and all those kinds of issues?

REESE: Well, that’s why you have review boards. The Zimbardo study that gets me is when he induced paranoia in college students.^17^ We’re paranoid enough without having it programmed! I don’t know how this passed the review board, but he was interested in a very important question, which is the relation between paranoia and loss of hearing. Because apparently, people, when they’re going blind, don’t become paranoid. But some elderly people who are becoming deaf, do. If two people are talking and glance over at the deaf person, the deaf person assumes they’re talking about them. Zimbardo hypnotized people and gave them the suggestion that they were losing their hearing. I don’t remember the rest of it, but he got lots of paranoid behavior. And I’m just so frightened by paranoia. There’s one reason I would never have passed on that study! But I think these ethical issues are terribly hard, whether they’re animals or people.

I think it’s a good idea to have students simulate review boards, either an animal care and use committee or a human research review committee, and pick some of these controversial studies. And then the students will get a feeling for how difficult these decisions are.

## I Adore Squirrels

OSU: A question that I’ve wondered about for a long time is why behavior analysts picked rats as lab animals in the first place? And why pigeons? Why haven’t we used animals like chipmunks? Why not woodpeckers? Or raccoons; they’re awfully smart.

REESE: One of the main reasons is that these domesticated animals take pretty well to captivity. Pigeons have been domesticated since at least 5,000 BC. We saw some of the old sites in Turkey when we were over there. They used to build recesses in the mud walls of some of the caves to keep the pigeons because their manure was great for fertilizing. And then, of course, rats have been selectively bred to be docile. I think those are the main reasons.

Another thing about pigeons is their vision is simply incredible. Their color vision may even be better than ours, they’re just super for any visual discrimination testing. And they work for pigeon food! They’re cheap. That’s another reason.

You pick animals because they mimic certain diseases or pathologies in people, but you also pick them because so much is known about them. And because rats have been used in medical research for so many years, we know a lot about rats and their husbandry and breeding and all the rest of that stuff. So that’s another reason researchers select particular animals to work with.

OSU: I hear you saying that once we have established a good genetic background, a good behavioral background, we continue using those species because we’re very familiar with them.

REESE: Well, that’s one reason. I did a lot of work on imprinting with chicks and ducklings—precocious birds—and then I wanted to go to a bird that rears its young in the nest for a while, to see if I’d get the same information about developing social attachments when working with birds that aren’t precocious. I use ringdoves in my teaching labs now instead of pigeons. That was a deliberate choice based on Danny Lehrman’s knowledge about ringdoves and their normal development.^18^ And again, ringdoves are domesticated and don’t mind reasonably small cages.

## It’s a Little Like the Skinner–Rogers Debate

OSU: There are some animal rights activists who say that because you’re restricting freedom in some way, that you shouldn’t do research at all with the animals in experimental settings. I’m wondering, have you had any trouble?

REESE: My first experience was at APA [American Psychological Association] in Anaheim, around 1982 or’83. There was a huge rally of animal rights people. This was PETA, People for the Ethical Treatment of Animals. They’re the gang that are behind all the laboratory break-ins. I walked up to a woman who was carrying one of the signs that psychologists were torturers and so forth, but she looked approachable. So, I went up and introduced myself, and I told her about Psychologists for the Ethical Treatment of Animals [PSYETA]. I said, “These are the psychologists who are trying to work for animals. Maybe you could get together with them.”

Anyway, she said, “Let me take you to…” and I’ve forgotten who it was, but their leader. She said, “This is Ellie Reese, and she works with animals, and she has some interesting…” He chimed in and said, “If you work with animals, I have nothing to say to you.” That was the end of that conversation.

Then, I managed to get on APA’s Committee on Animal Research and Ethics [CARE]. I asked if I could be the liaison to PSYETA because I knew that the PSYETA people wouldn’t talk with most of the people who were on CARE, and the animal experimentation people wouldn’t talk to the PSYETA people. So, I got myself made the liaison. And that was great. It was during the three-year period, when we were writing the APA guidelines for the ethical use and care of laboratory animals.^19^ I worked with the PSYETA people and was able to get the CARE committee to accept a number of their suggestions. We were the first guidelines to talk about the educational use of animals and problems there. So, it all depends who you’re talking with. I can talk with anybody in the PSYETA group. But with some of the others, it’s a little like the Skinner–Rogers debate. They’re not using language in the same way. They’re sort of two trains passing in the night.

OSU: I am a student representative on the Association for Behavior Analysis Executive Council. Some of the discussion in the past year has concerned whether certain kinds of advertisements should be allowed in the convention program book. Some of this was in response to an advertisement for Roger Ulrich’s book in the ABA conference program. I know that you’re very active in APA with some of these same issues, and I was just wondering how that’s been dealt with.

REESE: I would hate to see ABA blackball anything.^20^ I haven’t seen Roger’s book, but I assume it’s honest and scholarly. Have you seen it?

OSU: I only saw the advertisement. I think we have to look at what is being presented in the advertisement as well as the book. It was in the program guide this past year, and it condemned the use of animals in any way. And there were ABA members that were very upset because of the placement of the ad within…

REESE: [jumps in, reading from the ad] "*Rites of Life*: *A Book About the Use and Misuse of Animals on the Earth* [Ulrich, 1989]. Unless we surrender to other animals and watch and listen very carefully to them…" [stops reading] I don’t see anything the matter with the ad.

OSU: I guess there are several members that were very upset and felt that this was going against the work of trying to bring in more people from experimental analysis. So, the experimentalists were very upset by the …

REESE: Okay. There’s a sentence here that people could object to. He used to experiment on animals in labs, and he did perfectly terrible things. And I read all those studies. I’ve corresponded with Roger and met with him. I haven’t seen him recently, but during the Vietnam War he discovered peace and kindness. [Reese reading again from the ad] “The author is a researcher who used to experiment on animals in labs. However, he saw this was really torture based on the assumption that humans were somehow better than other forms of life.”

Well, a lot of Roger’s stuff was torture.^21^ Now, this sentence does not say that *all* animal research is torture. So, I still have no objection to it, basically.

Use whatever influence you’ve got as student representative on making sure ABA is dealing with real issues and doesn’t try to legislate opinions. And be careful about how to accept or reject ads.

## Combat the Conditions for Being Obscure

OSU: I read Karen Pryor’s book, *Don’t Shoot the Dog* (1984), while I was still teaching.^22^ It was fabulous. Very practical, realistic, and accurate. Pryor described great things that you could adopt in the classroom, too. In a similar vein, some of the professors here have been discussing the limited influence that behavior analysts have on the general public. And they suggest we might have more influence by publishing in *TV Guide*, *Red Book*, *Family Circle*, and periodicals of that nature, instead of trying to convince our colleagues in psychology and education with articles in traditional academic journals. What are your feelings about publishing in the popular press?

REESE: I think it’s a wonderful idea, if we learn how to talk in plain English. That’s one of our field’s greatest handicaps. A few people are simply marvelous. Paul Chance is one of them.^23^ He freelances for *Psychology Today*. He has a textbook out on learning that’s delightful. And whenever he does write an article about behavior analysis, it’s marvelous and we get super press. I think maybe the reason we don’t do it are the contingencies for promotion. Nobody counts anything but what comes out in some obscure journal. You don’t get many brownie points for publishing in *Sports Illustrated*, right?

What we do is wait for somebody to pick up on us, like the last issue of *Audubon Magazine* has a wonderful spread on Irene Pepperberg’s work with her parrot, Alex.^24^ And she has shown Alex to have all the cognitive abilities of the chimps and gorillas and dolphins and whales. And the reason she can demonstrate it is her teaching procedures are so marvelous. It’s just a neat article. They don’t say it’s behavior mod, unfortunately. But if we could get more articles like that out, it certainly would be marvelous.

ABA has a committee working on informing the public about behavior analysis. APA’s Division 25 is doing it by default. Maybe we should…hey! How about reducing somebody’s dues a certain proportion for every popular article they can get?

OSU: I like that idea. What do you think of Og Lindsley’s article in the summer issue of JABA discussing the use of plain English, both in our research and in our topical articles?^25^

REESE: Og has been arguing that point, which I think is an excellent one, for years. Og and I have been promoting this, but not publicly enough, for quite a while. And John Bailey is now joining the crew. What we need, I think, is to combat the conditions for being obscure. It’s really hard to get something published. I’m expecting a lot of grief from that Skinner article, whoever reviews it for AP [*American Psychologist*]. And I think that’s something people will read, whereas they won’t read things written in the reflexive and past tense and all these obscure constraints we put on ourselves.

 I don’t know that we can change that. Well, what you have to do is get a good editorial board. The first thing I submitted to a behavioral journal was the quail as a laboratory animal.^26^ I named the birds, Whetherby and Jennifer, and the paper was real chatty. Three people reviewed it. One said “reject” because the language was too casual or something. One loved the informality of the presentation, and one reviewer didn’t comment. The associate editor decided to go with it. But it could have been rejected on those grounds. So, that early reinforcement has kept me going, and I try to be as unstuffy as I can in writing. But it’s hard to encourage students unless you know the editors of what they’re going to send it to. The topic would be worth an editorial in one of our journals, at any rate. Maybe we’ll start a fund for people who do get stuff out in the popular press!

HEWARD: Ellie, this has been a fantastic session. Thank you so much for sharing your experience and knowledge with us today. We all look forward to seeing you at ABA next year.

REESE: Great. All right. Thank you so much. Good night. [students applaud].

## Data Availability

No data sets were generated or analyzed for this article.

## Footnotes

1 Students: Daniel Bullara, Rodney Cavanaugh, Lessie Cochran, Marie Cull, Gregg Drevno, Jim Dunne, Hua Feng, Frank Haden, Essie Johnson, Jonathan Kimball, Myra Middleton, April Miller, Mwajabu Possi, Bill Sweeney, Carolyn Talbert-Johnson, and Phillip Ward. Faculty: John Cooper, John Eshleman, Tim Heron, and Bill Heward.

2 The paper was never published. A PDF of the manuscript can be requested from Mount Holyoke College Archives and Special Collections https://lits.mtholyoke.edu/about-lits/service-desks/archives-special-collections.

3 The teleconference took place nearly 35 years ago. Terms used to identify people with intellectual disabilities and some research and applied practices (e.g., extended extinction conditions) of that era are inappropriate and unethical today. We’ve changed terminology to reflect contemporary usage.

4 “Ellen Pulford was a student when her future husband, Mount Holyoke professor Thomas W. Reese, established one of the first two introductory psychology laboratory courses built upon the Keller and Schoenfeld model. Both laboratories opened simultaneously at Columbia College and Mount Holyoke College in 1946. Ellie became a research assistant in 1948, and she became Mrs. Reese in 1949.” (Poulson, 1998, p. 1).

5 See Corbin et al. (1956), Jensen et al. (1950), and Reese, E. P. et al. (1953).

6 Individual students are not identified by name. Some questions are slightly edited combinations of questions, and not all students’ questions are included in the article.

7 In the B. F. Skinner’s Contributions to Animal Welfare paper, Reese (1991) cites a number of studies of shaping the acceptance of needles or catheters (for obtaining samples or introducing drugs) and one of teaching vervet monkeys to urinate in a beaker.

8 See Hager et al. (1980) and Reese (1981).

9 See Carr and Durand (1985) and Durand and Carr (1987).

10 See Malott (1988).

11 Founded in the United Kingdom in 1942, the Oxford Committee for Famine Relief (Oxfam) campaigned for food supplies to be sent through an allied naval blockade to starving women and children in enemy-occupied Greece during the Second World War (https://www.oxfam.org/en/our-history). In 1995, a group of independent non-governmental organizations formed Oxfam International, whose 21 members work with partners in 79 countries to fight hunger, inequality, and injustice (https://www.oxfam.org/en/what-we-do/about). For free access to Oxfam educational resources and simulation games on food inequality, fair trade, climate change, gender justice, and women’s rights, go to https://policy-practice.oxfam.org/keyword/simulation-games/. For examples of research on role-playing games, see Harris et al. (2015) and Krain and Shadle (2006).

12 *A New Learning Environment: A Case for Learning* (Cohen & Filipczak, 1989). See review by Ellis (1992).

13 Note that freeloading is in quotes. Freeloading is a technical term, without the value-laden, negative political connotations of Reagan’s “welfare queens.” From Reese (1991): “Rats (Joffe, Rawson, & Mulick, 1973) and rhesus monkeys (Mineka, Gunnar, & Champoux, 1986) who earn their food and water in a contingent operant environment subsequently exhibit less fear than controls in novel, stressful situations. In addition, forced ‘freeloading’ as opposed to working for food, can impair subsequent learning, both to obtain food (Welker, 1976) and to learn a shuttle box escape response (Goodkin, 1976).” (p. 22).

14 At this point, Ellie said, “Kathy Dyer, Dunlap, and Winterling in JABA, 1990, volume 23. Starts on page 515, and she’s got some other references there. Mike Cataldo has one about kids who are in a pediatric intensive care unit. That’s an old one: JABA, 1979, volume 12, page 83 to 97. There’s a Koegel article in JABA, 1987, volume 20, starts on page 243. And there’s Datillo and Rusch in the *Journal of the Association for Persons with Severe Handicaps*, 1985, volume 10, pages 194 to 199. And Parsons has two in JABA, 1990. That’s a pretty good start.”.

15 The atrocities of the holocaust led social psychologist Stanley Milgram (1933–1985) to investigate the extent to which people would be induced by an authority figure to harm fellow humans. In an infamous experiment, beginning in 1961 at Yale University (Milgram, 1963), subjects were deceived that they were participating in an investigation of the effects of punishment on learning. Milgram’s actual question was the extent to which people would administer punishment—in this case, delivering electric shocks of increasing intensity—when directed to do so by a scientist in a lab coat. There was no shock; the supposed recipients were in another room and pretended to cry out in pain. Some subjects delivered shocks to the point that it would have been lethal. This experiment provided impetus for changing rules of ethics for psychological research.

16 Social psychologist Philip G. Zimbardo (1933–2024) is perhaps best known for an extended role-play study known as the Stanford Prison Experiment (Zimbardo et al., 1971). This investigation represented the opposite side of Milgram’s coin by examining how possessing authority might induce antisocial behavior. What was meant to be a 2-week study was suspended after 6 days because college students acting as guards became so abusive that some of those playing prisoners suffered acute psychological trauma.

17 Zimbardo et al. (1981). The abstract of that study states, “The development of paranoid reactions was investigated in normal people experiencing a temporary loss of hearing. In a social setting, subjects made partially deaf by hypnotic suggestion, but kept unaware of the source of their deafness, became more paranoid as indicated on a variety of assessment measures. The results support a hypothesized cognitive-social mechanism for the clinically observed relationship between paranoia and deafness in the elderly.”

18 Danny Lehrman (1919–1972), an American psychologist and ornithologist, was founder and director of the Institute of Animal Behavior at Rutgers, and a member of the National Academy of Sciences, which said that “he influenced a whole generation of students in animal behavior in this country and abroad.” Source: Wikipedia.

19 The most recent version of APAs *Guidelines for Ethical Conduct in the Care and Use of Nonhuman Animals in Research* (2022) can be downloaded at https://www.apa.org/science/leadership/care/animal-guide.pdf

20 The Association for Behavior Analysis changed its name to the Association for Behavior Analysis International in 2008.

21 Professor Reese did not cite specific examples, but we assume she was referring to studies such as Ulrich et al., (1962, 1965, 1969).

22 Karen Pryor (1932–2025) was internationally renowned for teaching about the power and effective use of positive reinforcement. “She changed people’s and animals’ lives by making positive reinforcement and welfare-based animal care THE way to approach teaching and training” (Ramirez, 2025, p. 30). *Don’t Shoot the Dog* was translated into 27 languages and remains in print today (Pryor, 2002).

23 Paul Chance (1941–2021) was an exceptionally clear and effective writer. For examples of his writing for the benefit of teachers, students, and behavior analysts, see Chance (2007, 2008, 2013).

24 We were unable to find the *Audubon Magazine* article, but books by Pepperberg (2002, 2021) detail her work with her parrot, Alex.

25 Lindsley (1991).

26 See Reese, E. P. and Reese, T. W. (1962).
